# Osimertinib Improves the Immune Microenvironment of Lung Cancer by Downregulating PD-L1 Expression of Vascular Endothelial Cells and Enhances the Antitumor Effect of Bevacizumab

**DOI:** 10.1155/2022/1531353

**Published:** 2022-06-23

**Authors:** Xuejun Xiao, Yang Wu, Fang Shen, Yusufu MuLaTiAize, Nabi Xinhua

**Affiliations:** Department of Pharmacology, Xinjiang Medical University, Urumqi 830054, China

## Abstract

**Objective:**

To investigate the effect and mechanism of osimertinib combined with bevacizumab on lung cancer through cell and transplanted tumor animal experiments and to provide theoretical basis for further clinical trials.

**Methods:**

Immunohistochemistry was used to detect the expression of PD-L1 in tumor vessels of nonmetastatic lung adenocarcinoma and metastatic lung adenocarcinoma. At the same time, the expression of CD8 and FoxP3 in tumor tissue was detected. qRT-PCR was used to detect the effect of osimertinib on PD-L1 expression in HUVECs. The expression levels of p-Akt and p-ERK in HUVECs treated with osimertinib were analyzed by Western blot. AKT was blocked by AKT specific inhibitor Ly294002 to analyze the expression of PD-L1 in HUVECs. An animal model of transplanted tumor was constructed to analyze whether osimertinib could enhance the antitumor effect of bevacizumab.

**Results:**

PD-L1 was highly expressed in vascular endothelial cells of metastatic lung cancer. FoxP3 was highly expressed in metastatic lung adenocarcinoma, while CD8 expression was low. Osimertinib inhibits PD-L1 expression in endothelial cells. Mechanism studies have shown that osimertinib inhibits PD-L1 expression in endothelial cells through the AKT/ERK pathway. Osimertinib inhibited endothelial cell PD-L1 expression, increased CD8^+^T cell infiltration, inhibited tumor growth, and enhanced the tumor effect of bevacizumab.

**Conclusion:**

Osimertinib can significantly increase the killing ability of bevacizumab against tumor. Osimertinib can improve the tumor microenvironment and enhance the antitumor effect of bevacizumab by reducing the expression of PD-L1 in tumor blood vessels.

## 1. Introduction

Lung cancer is one of the most deadly malignant tumors [1]. Nearly 85% of all lung cancer cases are non-small-cell lung cancer (NSCLC) [2]. The early symptoms of NSCLC patients are not obvious, leading to the diagnosis when most patients are already in the middle and late stage. Chemotherapy and radiotherapy are the main methods for the treatment of advanced NSCLC. However, due to the high degree of metastasis of lung cancer, the treatment effect is not ideal [3]. Therefore, the exploration of effective treatment methods has become the focus of people's attention in recent years [4]. The occurrence and development of malignant tumor are closely related to immune dysfunction. In recent years, tumor immunotherapy has made great progress in the treatment of NSCLC. Programmed death-1 (PD-1) and its ligand PD-L1 play an important role in tumor immune evasion and formation of the tumor microenvironment [5].

PD-L1 is expressed in a variety of tumors, especially NSCLC [6], melanoma, kidney cancer, prostate cancer, liver cancer, leukemia, multiple myeloma, etc. [7, 8]. The expression of PD-L1 can induce the production of multiple proinflammatory factors. PD-L1 is regulated by oncogenes and tumor suppressor genes. For example, the expression of PD-L1 is inhibited by tumor suppressor gene PTEN, which belongs to autoimmunity. Parsa et al. found that knockout of PTEN in glioma can regulate the expression of PD-L1 at the transcriptional level. Its mechanism of action is to activate the downstream mTOR-s6k1 signaling pathway of PI3K/AKT and ultimately increase the expression of PD-L1 [6, 9].

In recent years, personalized molecular targeted therapy has achieved great success. For NSCLC with EGFR-sensitive mutations (exon 19 deletion (E19del) and exon 21 point mutation (E21L858R), treatment with first-generation or second-generation EGFR-TKIs can significantly improve the prognosis of patients compared with traditional chemotherapy [10]. Osimertinib is a third representative dermal growth factor receptor tyrosine kinase inhibitor that has been shown to be effective in prolonging survival and reducing the risk of suffering and disease in patients with non-small-cell lung cancer [10]. Osimertinib is widely used in the treatment of non-small-cell lung cancer [10–13]. However, its mechanism of action needs further study [14]. Bevacizumab is a humanized recombinant monoclonal antibody against the vascular endothelial growth factor IgG1 [15]. By blocking the binding of the vascular endothelial growth factor to its receptor, the angiogenic activity of the vascular endothelial growth factor was inhibited, thus exerting antitumor effect [16]. Bevacizumab reduces tumor resistance to conventional chemotherapy or targeted drugs [17].

Studies have shown [18] that PD-L1 plays an important role in tumor evasion. Whether osimertinib affects the tumor immune microenvironment is unknown. Therefore, this study studied the inhibitory effect of osimertinib on tumor growth in human lung cancer-bearing mice from the perspective of the immune microenvironment. At the same time, the antitumor effect of the combination of osimertinib and bevacizumab was analyzed to provide a new theoretical basis for future clinical research on cancer.

## 2. Methods

### 2.1. Tumor Tissue Collection of Metastatic Lung Cancer and Nonmetastatic Lung Cancer

25 surgical specimens of metastatic and nonmetastatic NSCLC were collected from our hospital from April 2018 to January 2021. All patients underwent radical surgery, histologically diagnosed as NSCLC, and did not receive any preoperative antitumor therapy. The diagnostic basis of specimens collected in this study is CSCO Guidelines on the diagnosis and treatment of primary bronchogenic carcinoma. The diagnosis was independently evaluated by two pathologists. All patients signed informed consent. The study was approved by the Ethics Committee of Xinjiang Medical University.

### 2.2. Immunohistochemistry of Tumor Tissue

Paraffin sections were taken from the tissues to prepare continuous sections with a thickness of about 4 *µ*m. The slices were dewaxed and hydrated and placed in disodium hydrogen phosphate citric acid buffer. The antigen was repaired with boiling water bath under high pressure, and the reaction time was 2 min. The slices were removed and cooled naturally and then soaked in 3% hydrogen peroxide solution for 15 min. Then slice and place in a wet box, and block with normal goat blood at room temperature for 20 minutes. Diluted CD8, FoxP3, and PD-L1 antibodies were dropped, respectively. Their dilution ratio was 1 : 1000 and they were placed in a wet box at 4°C for overnight incubation. The diluted biotin-labeled secondary antibody was dropped, diluted at 1 : 5000, and placed in a wet box at room temperature for 20 min. DAB was used for color detection. When the observed parts were brownish yellow or brown, the observed parts were washed with running water for 3 times, 5 min each. Hematoxylin was redyed for 5 min and washed with running water. They were dehydrated, transparent, sealed with neutral gum, observed, and stained under a microscope.

### 2.3. qRT-PCR

RNA was extracted according to the instructions of the TRIzol kit. The extracted RNA was stored at −80°C. Reverse transcription and real-time qPCR were performed according to the instructions of TAKARA Prime Script™ RT Master Mix and SYRB Premix Ex Taq™ kit, respectively. Real-time qPCR amplification was used for fluorescence amplification, and GAPDH was used as a housekeeping gene. The reaction strip was prevariable at 95°C for 3 min, denaturation at 95°C for 10 s, annealing at 58°C for 15 s, and extension at 72°C for 20 s, with a total of 40 cycles. GAPDH primers forward: 5′-GCACCGTCAAGGCTGAGAAC-3′ and reverse: 5′-TGGTGAAGACGCCAGTGGA-3′. The relative expression of the target gene was analyzed by the 2^−ΔΔCt^ method.

### 2.4. Western Blot

Cells or tissues are lysed (with the addition of protease and phosphatase inhibitors), and proteins are extracted. The protein concentration was determined by the BCA method, and the 5x loading buffer was mixed. It was boiled for 3 min and cooled quickly in an ice bath. The loading amount was 30 *μ*g per lane. After polyacrylamide gel electrophoresis, it was transferred to PVDF membrane and blocked with calf serum albumin for 2 h. The corresponding primary antibody was added and incubated at 4°C overnight (GAPDH is the internal reference). After washing, the cells were incubated with secondary antibodies and chemiluminescence was developed. ImageJ was used to analyze the gray value detection and analysis of each target protein.

### 2.5. Subcutaneous Xenograft Model

Cells were cultured in the RPMI1640 medium (containing 10% fetal bovine serum and 1% penicillin-streptomycin) and cultured in an incubator at 37°C, 5% CO_2_, and 90% humidity, and the cells adhered. The cells in the logarithmic growth phase were taken, digested, and counted, and 2 × 10^6^/mL cells were inoculated subcutaneously in the flank wall in front of the right hindlimb of mice. Tumor volumes were measured at least twice a week after inoculation and during dosing. Mice were randomly divided into 3 groups: (1) control, (2) osimertinib, and (3) osimertinib + bevacizumab (*n* = 6); osimertinib: 2.5 mg/kg/d and bevacizumab: 5 mg/kg twice a week. Method of administration: osimertinib was administered by gavage daily, and bevacizumab was injected intraperitoneally twice a week. Tumor growth curves were drawn after inoculation and during dosing. All nude mice were euthanized, and whole tumor excised sections were biopsied and placed in 4% paraformaldehyde solution. The remainder was frozen in liquid nitrogen. Animal experiments in this study followed animal welfare and ethical requirements.

### 2.6. Immunofluorescence

Section dewaxing, hydration, antigen retrieval, and blocking steps were the same as for immunohistochemistry. After blocking, the blocking solution was washed, and the diluted primary antibody working solution of CD8 and FoxP3 was added dropwise. After incubation at room temperature, the cells were placed in a humidified chamber at 4°C overnight. The primary antibody was washed, and the corresponding fluorescent secondary antibody was added dropwise and incubated at room temperature for 60 min in the dark. DAPI was added dropwise to completely cover the tissue to counterstain nuclei for 8 min and washed with PBS. The liquid around the tissue was wiped dry, covered with a coverslip, and photographed by a confocal microscope.

### 2.7. Statistical Analysis

Statistical analysis was performed using SPSS 20.0 software. Data are expressed as the mean ± standard deviation. The *t*-test was used for comparison between the two groups. *P* < 0.05 was considered to be statistically significant.

## 3. Results

### 3.1. PD-L1 Is Highly Expressed in Vascular Endothelial Cells in Metastatic Lung Adenocarcinoma Tumor Tissue

**Figure 1:**
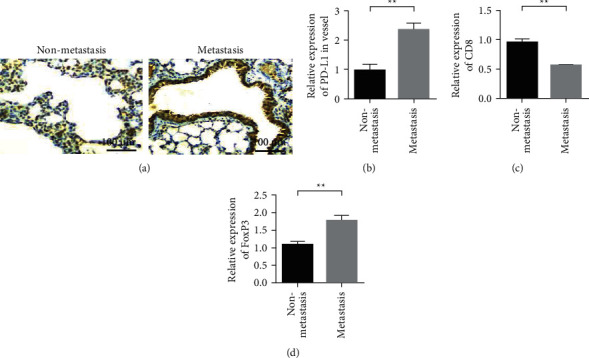
PD-L1 is highly expressed in vascular endothelial cells in metastatic lung cancer tumor tissue. (a) Immunohistochemical staining images of endothelial cells with high PD-L1 expression in metastatic and nonmetastatic lung cancers (*n* = 25). (b) Statistical results of PD-L1 expression. (c) Immunohistochemical staining results of CD8 in metastatic and nonmetastatic lung cancer tissues. (d) FoxP3 immunohistochemical staining results in metastatic and nonmetastatic lung cancer tumor tissues.  ^*∗*^ ^*∗*^*P* < 0.01.

**Figure 2:**
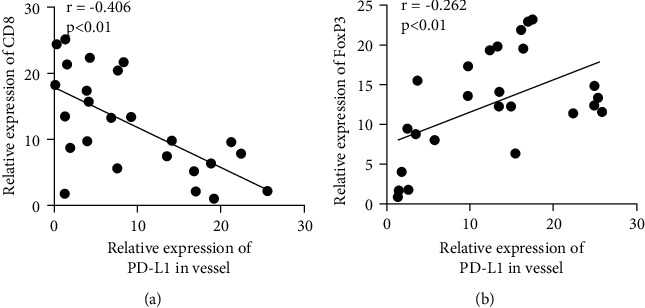
Correlation analysis of coexpression of PD-L1 with CD8 and FoxP3 in metastatic lung cancer tumor tissue. (a) Correlation analysis of PD-L1 and CD8 coexpression in metastatic lung cancer tumor tissue. (b) Correlation analysis of PD-L1 and FoxP3 coexpression in metastatic lung cancer tumor tissue.  ^*∗*^ ^*∗*^*P* < 0.01.

This study focused on the expression of PD-L1 in vascular endothelial cells in tumor tissue. Immunohistochemical staining showed that compared with the tumor tissues of nonmetastatic lung cancer patients, the expression of PD-L1 in vascular endothelial cells in the tumor tissues of patients with metastatic lung cancer was increased (Figures 1(a) and 1(b)). CD8 was mainly expressed in the cytoplasm, and the positive rate of CD8 in metastatic lung cancer tissues of NSCLC was significantly lower than that in nonmetastatic tumor tissues (Figure 1(c)). Immunohistochemical results showed that FoxP3 was highly expressed in metastatic lung cancer tumor tissues (Figure 1(d)). The correlation analysis of PD-L1 coexpression with CD8 and FoxP3 in metastatic lung adenocarcinoma tumor tissue showed that PD-L1 and CD8 were negatively correlated with coexpression in lung cancer tumor tissue (Figure 2(a)). In metastatic lung adenocarcinoma tumor tissue, PD-L1 and FoxP3 showed a positive correlation with coexpression (Figure 2(b)).

### 3.2. Osimertinib Inhibits the Expression of PD-L1 in Endothelial Cells

**Figure 3:**
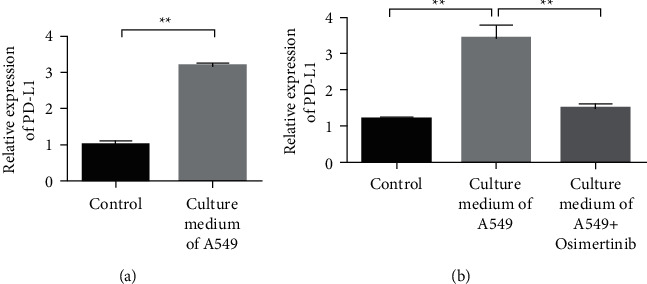
Osimertinib inhibits PD-L1 expression on endothelial cells. (a) The expression of PD-L1 was detected after HUVECs were treated with the tumor cell (CM) supernatant for 48 h. (b) Osimertinib inhibits the expression of PD-L1 in HUVECs.  ^*∗*^ ^*∗*^*P* < 0.01.

The complex mechanism of tumor metastasis lies in the extensive interactions that occur between tumor cells and various host cells. Tumor cells regulate the function and state of vascular endothelial cells through a variety of mechanisms. This study firstly analyzed the effect of the lung cancer cell culture medium on HUVECs. The expression of PD-L1 was detected after HUVECs were treated with tumor cell (CM) supernatant for 48 h. The results showed that CM could upregulate the expression of PD-L1 in endothelial cells (Figure 3(a)). Subsequently, we examined the effect of lung cancer-targeted drug osimertinib on the expression of PD-L1 in endothelial cells. The experiment was divided into three groups: (1) control, (2) lung cancer cell culture medium supernatant, and (3) lung cancer cell culture medium supernatant + osimertinib. The experimental results showed that the expression of PD-L1 was decreased in the CM + osimertinib-treated group compared with the CM-treated group (Figure 3(b)).

### 3.3. Osimertinib Inhibits the Expression of PD-L1 in Endothelial Cells through the AKT/ERK Pathway

**Figure 4:**
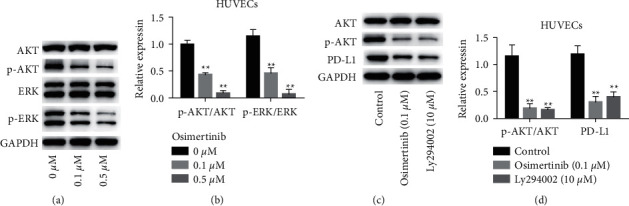
Osimertinib inhibits the expression of PD-L1 in endothelial cells via the AKT/ERK pathway. (a) Western blot analysis showed that the expressions of p-AKT and p-ERK were decreased in osimertinib-treated HUVECs. (b) Statistical analysis results of p-AKT/AKT and p-ERK/ERK. (c) Blocking AKT can reduce the expression of PD-L1. (d) Statistical results of each group of proteins.  ^*∗*^ ^*∗*^*P* < 0.01.

Studies have reported that the AKT signaling pathway mediates the expression of PD-L1 in cells. Western blot was used to analyze the expressions of p-AKT and p-ERK in osimertinib-treated HUVECs. The results showed that, compared with the control group, the expressions of p-AKT and p-ERK in HUVECs were decreased after osimertinib treatment (Figures 4(a) and 4(b)). Further, we used the AKT-specific inhibitor Ly294002 to verify whether blocking AKT could reduce the expression of PD-L1. The results showed that the expression of p-AKT was decreased in HUVECs treated with osimertinib and Ly294002. At the same time, PD-L1 expression was also decreased (Figures 4(c) and 4(d)). The above experimental results indicate that osimertinib inhibits the expression of PD-L1 in endothelial cells through the AKT/ERK pathway.

### 3.4. Osimertinib Inhibits the Expression of PD-L1 in Endothelial Cells and Inhibits Tumor Growth

**Figure 5:**
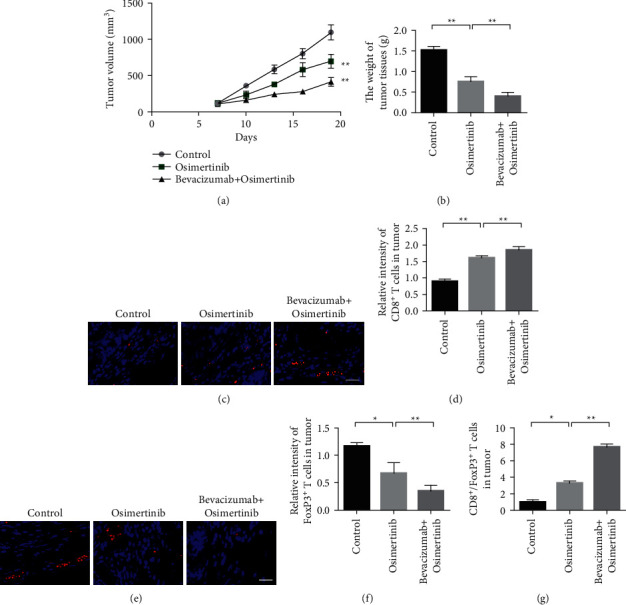
Osimertinib inhibits PD-L1 expression in endothelial cells and inhibits tumor growth. (a) C57BL/6 mice were injected with 1 × 10^6^ B16 cells, and tumors grew. The tumor growth curve of each treatment group (*n* = 6 per group). (b) Tumor weight in each treatment group. (c) Representative images of immune cell markers (CD8^+^ T cells) in B16 tumor tissue. (d) Quantification of CD8^+^ T cells in B16 tumors. (e) Representative images of immune cell markers (FoxP3^+^ T cells) in B16 tumor tissue. (f) Quantification of FoxP3^+^ T cells in B16 tumors. (g) Statistics of CD8/FoxP3 ratio in tumor tissue.  ^*∗*^*P* < 0.05,  ^*∗*^ ^*∗*^*P* < 0.01.

This study further investigated the ability of osimertinib to increase the antitumor effect of bevacizumab. C57BL/6 mice were injected with B16 cells. Mice were divided into 3 groups (*n* = 6 per group) and treated with either osimertinib or osimertinib + bevacizumab. The changes of tumor volume and tumor weight of transplanted tumor nude mice in each treatment group are shown in the figure. After administration, the tumor volume and tumor weight of the osimertinib combined with the bevacizumab group were significantly smaller than those of the osimertinib single-agent group (Figures 5(a) and 5(b)). The results of CD8^+^T cell immunofluorescence staining showed that the CD8^+^T cell content in the tumor tissue of osimertinib combined with the bevacizumab group was higher than that of the osimertinib single-agent group (Figures 5(c) and 5(d)). The results of FoxP3^+^T cell immunofluorescence staining showed that the FoxP3^+^T cell content in the tumor tissue of osimertinib combined with the bevacizumab group was lower than that of the osimertinib single-agent group (Figures 5(e) and 5(f)). Statistics of CD8/FoxP3 ratio in tumor tissue showed that the infiltration rate of CD8^+^ T cells was increased after the combination of osimertinib and bevacizumab, while the infiltration rate of FoxP3^+^ T cells was decreased (Figure 5(g)).

## 4. Discussion

Tumor microenvironment plays a central role in the development and progression of lung cancer [19]. Innate and adaptive immune cells in the tumor microenvironment have protumor and antitumor effects. Tumor cells overexpress immune checkpoint molecules to escape the surveillance and killing of immune cells, thus promoting tumor growth [20]. Further study on the immune regulation function of immune cells is helpful to improve the efficacy of immunotherapy and find new therapeutic targets [21, 22].

CD8^+^T cells play an important role in the tumor immune microenvironment, and their invasion level can affect the efficacy of tumor immunotherapy [23]. It has been reported that the infiltration rate of CD8^+^T cells in NSCLC tissues is about 68.9% [24]. In this study, the infiltration rate of CD8^+^T cells in metastatic NSCLC lesions was lower than that in the primary lesions. The decrease of CD8^+^T cell infiltration may be one of the important reasons for NSCLC metastasis. However, this study only observed the infiltration of CD8^+^T cells in tumor tissues, which could not represent the whole immune microenvironment in tumor area. The changes of CD3^+^, CD4^+,^ and CD8^+^T cells in blood could be further analyzed. Fork head box protein 3 (FoxP3)^+^ regulatory T cells (Treg) are important immune regulatory cells, which play an important role in the development of many kinds of malignant tumors. A lot of studies prove, FoxP3 is expressed in a variety of tumors such as colon cancer, pancreatic cancer, liver cancer, bladder cancer, breast cancer, melanoma, lung cancer, prostate cancer, and glioma. FoxP3 is located in Treg and/or tumor cells and is one of the factors of poor prognosis of tumor [25–29]. Reduction of FoxP3+T was observed after osimertinib administration. Meanwhile, fewer FoxP3+T cells were observed after osimertinib was combined with bevacizumab. These results suggest that FoxP3^+^T, a negative immunoregulatory cell, is involved in the malignant progression of lung cancer. Osimertinib can inhibit negative immune regulation.

Vascular endothelial growth factor (VEGF) plays an important role in angiogenesis and is essential for the survival of endothelial cells in tumors [30, 31]. Bevacizumab can reduce VEGF levels and block neovascularization, temporarily normalize tumor tortuous blood vessels, improve tumor oxygenation and reduce interstitial fluid pressure, and restore drug delivery to the tumor. This may make tumor cells more sensitive to EGFR-TKIs [32]. This study showed that osimertinib inhibited the AKT/ERK signaling pathway and then inhibited the expression of PD-L1 in endothelial cells. It can be observed from the tumor growth curve that the synergistic tumor inhibition effect of bevacizumab is more significant than the single dose double tumor inhibition effect. It is suggested that an appropriate dose of osimertinib combined with antivascular therapy may bring more benefits than increasing the dose of osimertinib alone. The combination of bevacizumab improved the microenvironment in tumor tissue and enhanced the inhibitory effect of osimertinib, thus killing tumor cells more effectively.

In this study, although bevacizumab inhibited tumor angiogenesis, it improved the tumor internal environment and thus improved oxygen supply in tumor tissues. This is also in line with the normalization phenomenon of blood vessels in antiangiogenic therapy proposed by Jain et al. [33, 34].

There are also some deficiencies in this study. Protein kinase B, or AKT, plays an important role in cell survival and apoptosis. The PI3 kinase-AKT pathway is a classical signaling pathway. Many kinase inhibitors inhibit AKT activation when they inhibit PI3 kinase. Total AKT was detected in this study without further distinction between AKT1, 2, 3. The role and differences of AKT subtypes will be analyzed in subsequent experiments. In this study, we found that osimertinib inhibited ERK phosphorylation. In subsequent experiments, we will analyze the role and differences of ERK subtypes in this process.

## 5. Conclusion

In conclusion, this study confirmed that the 3rd generation EGFR-TKI osimertinib has a strong tumor suppressive effect on lung adenocarcinoma transplanted tumor through cell experiment and animal experiment of transplanted tumor. Bevacizumab can significantly increase the killing ability of osimertinib against lung adenocarcinoma transplanted tumor, and the two have a synergistic effect. The synergistic effect of bevacizumab and osimertinib is achieved by reducing PD-L1 expression in tumor blood vessels, improving tumor microenvironment, and enhancing the function of immune cells. This study provides theoretical basis for further clinical trials.

## Data Availability

The data used to support the findings of this study are available from the corresponding author upon request.
